# Evaluation of Oral Squamous Cell Carcinoma Patients Using Polymerase Chain Reaction Regarding the Prevalence of Human Papillomavirus Types 16 and 18

**DOI:** 10.7759/cureus.51938

**Published:** 2024-01-09

**Authors:** Prabhakar A Jeergal, Vasanti A Jeergal, Samreen Fatma, Arpanna Singh, Rohit Sharma, Madhuri S Sale

**Affiliations:** 1 Department of Oral Pathology, Sudha Rustagi College of Dental Sciences, Faridabad, IND; 2 Department of General Medicine, Srinivas Institute of Medical Sciences and Research Centre, Mangaluru, IND; 3 Department of Oral Pathology and Microbiology, Mithila Minority Dental College and Hospital, Darbhanga, IND; 4 Department of Pedodontics and Preventive Dentistry, Community Health Centre, Health and Family Welfare Uttar Pradesh, Ghazipur, IND; 5 Department of Oral and Maxillofacial Surgery, School of Dental Sciences, Sharda University, Greater Noida, IND; 6 Department of Oral Pathology and Microbiology, Bharati Vidyapeeth Deemed to be University Dental College and Hospital, Sangli, IND

**Keywords:** human papillomavirus, retromolar region, pcr, oral squamous cell carcinoma, hpv 18, hpv 16

## Abstract

Background: Epstein-Barr virus, human papillomavirus (HPV), and herpes simplex virus type 1 (HSV-1) are examples of viruses that have been associated with the development of oral squamous cell carcinoma (OSCC). These viruses can infect various epithelial tissues in the human body. The use of incredibly accurate cellular biology techniques, such as the polymerase chain reaction (PCR), which permits the rapid identification of viruses following infection, has increased. The parameters of human head and neck oncology have been widened.

Aim: In this study, using the PCR, the presence of HPV variants such as HPV 18 and HPV 16 in patients with OSCC was assessed.

Materials and methods: Tissue specimens were obtained from clinically presumed OSCC individuals taken as cases, and tissues from the retromolar region were obtained from people who experienced an operation for partially and completely impacted tooth and taken as controls. The study included 80 samples divided into two separate categories: case category (n = 40) = OSCC-diagnosed individuals; control category (n = 40) = controls with a comparable age. For verification of the diagnosis, a specimen of the tissue has been processed and sections have been stained and inspected for standard hematoxylin and eosin stain. Deoxyribonucleic acid (DNA) was extracted from the leftover histopathologically verified tissue specimens and then exposed to PCR for the assessment of HPV infiltration.

Results: It was observed in this research that 22 cases out of 40 cases of OSCC were found positive for HPV-DNA. While 12 out of 40 age-matched healthy controls were found positive for HPV-DNA. Out of 40 cases of OSCC, 12 cases were found positive for HPV 16. While six out of 40 age-matched healthy controls were found positive for HPV 16. Six cases out of 40 cases of OSCC were found positive for HPV 16. While two out of 40 age-matched healthy controls were found positive for HPV 18. Four cases out of 40 cases of OSCC were found positive for HPV 16. While four out of 40 age-matched healthy controls were found positive for HPV 16 and HPV 18. On carrying out statistical analysis, the variation between the two categories was non-meaningful statistically (p = 0.662). However, the prevalence was greater in the case (OSCC) subgroup.

Conclusion: When evaluated against controls in the current investigation, OSCC cases had a greater level of HPV expression and a greater proportion of HPV 16 positives. However, there was no statistically noteworthy change.

## Introduction

Among the top 10 most prevalent cancers in the world, cancer in the region of the head and neck affects more than 500,000 people annually [1,2]. More than 90% of oral cavity cancers are squamous cell carcinomas (SCC), which are the most common type. It ranks as the sixth most common malignant tumor globally [3,4]. Risk variables for the occurrence of oral squamous cell carcinoma (OSCC) include alcohol, smokeless tobacco products like betel quid, pan masala, and gutka, as well as smoking cigarettes [5,6]. Other risk variables like viruses that may contribute to OSCC establishment include the Epstein-Barr virus, the human papillomavirus (HPV), and herpes simplex virus 1 (HSV-1) [7,8]. Smaller deoxyribonucleic acid (DNA) viruses known as HPVs invade a variety of epithelial tissues of humans.

In accordance with their capacity to cause cancer, more than 130 different HPV kinds have been recognized and are divided into modestly dangerous and extremely dangerous categories [9-11]. Squamous cell carcinomas of the oropharynx (OPSCCs) with HPV are a separate clinical condition with significantly better survival than OPSCCs without HPV, which are frequently linked to both alcohol intake and tobacco addiction [12,13]. There is mounting proof that extremely dangerous HPV varieties, primarily HPV 16/18, as well as OSCC, are causally linked. Multiple research studies have demonstrated that HPV is linked to an increased probability of the oral cavity, irrespective of the consumption of alcohol and cigarettes. Due to its identification in oral dysplastic disorders and oral malignancies, this connection holds true for both HPV 16 and HPV 18 [14,15].

Due to challenges in deciphering research that showed rates of prevalence that varied from 0% to 100%, the exact nature of the link involving HPVs and OSCC is still unknown. Interpretation is additionally challenging due to the various demographics and tests' differing levels of sensibility in identifying viral DNA [15].

The total amount of relationships with sexual partners over the course of a person's entire life is thought to be a vital risk indicator for the possibility of HPV-linked SCC of the head and neck. HPV is an infection that is transmitted through sexual contact. In case-control investigations, the likelihood of developing a malignancy with HPV positive was shown to be twice as prevalent in people who admitted to having between a single and five oral sex relationships in the course of their lives and five times higher in people who admitted having six or more than six partners [16].

The frequencies of HPV positives in malignancies at different locations in the healthy normal mucosa (HNR) show an extensive spectrum of variance. Tonsil and buccal mucosa cancers have the greatest rates, subsequently followed by tongue and throat malignancies [17]. Dysplasia in the tissues of the oral cavity and OSCC are more likely to be related to infection caused by HPV, specifically subtypes 16 and 18, as opposed to the condition of healthy oral mucosa (HOM), reported by previously published meta-analysis focusing on HPV infestation of the mucous membrane of the mouth [18].

The use of exceptionally precise cellular biology technologies, including polymerase chain reaction (PCR), enables the identification of viruses within a short duration after infection. It can also help in the identification of viral infection in the period between infection by virus and the appearance of symptoms of actual disease. It has expanded the boundaries of human oncology. Utilizing PCR, the presentation of HPV variants like HPV 18 and HPV 16 in patients affected by OSCC was evaluated in this research.

## Materials and methods

Source of information

The present study was carried out at the Srinivas Institute of Medical Sciences and Research Centre, Karnataka from May 2021 to June 2022. In the present investigation, participants were between the ages of 20 and 60 years with 24 males and 16 females. Tissue specimens were obtained from clinically presumed OSCC individuals taken as cases, and tissues from the retromolar region were obtained from people who experienced an operation for partially and completely impacted tooth and taken as controls. The study included 80 samples divided into two separate categories: case category (n = 40) = OSCC-diagnosed individuals; control category (n = 40) = controls with a comparable age.

Inclusion criteria for the case group include individuals clinically diagnosed with OSCC, age-matched with the control group, willingness to participate in the study, and availability of tissue specimens for collection. Exclusion criteria for the case group include individuals with other types of oral or systemic cancers and patients with a history of prior treatment for OSCC.

Inclusion criteria for the control group include healthy individuals without a diagnosis of OSCC, age-matched with the case group, willingness to participate in the study, and availability of tissue specimens from the retromolar region due to impacted tooth surgery. Exclusion criteria for the control group include individuals with a history of oral or systemic cancers, individuals with any oral or neck abnormalities that could affect the study, and patients with a history of prior treatment for oral cancers.

Study methodology

Part of the tissue was processed and sections were stained and examined for routine hematoxylin and eosin to confirm the diagnosis. From the remaining part of the histologically proven tissues, DNA extraction was done and subjected to PCR for the evaluation of HPV‑positive samples.

Gathering of samples

Both OSCC individuals and healthy age-matched controls provided specimens for collection. Tissue specimens were procured, placed in a tiny zip lock pouch, submerged in liquid nitrogen, and then preserved at -20°C until needed.

Procedure for extracting DNA from new specimens of tissue

The process of dehydration of the tissue specimen was accomplished by adding 1 ml alcohol centrifuging the entire mixture for 30 minutes and then eliminating the supernatant. The pellet was then vortexed and immersed in 500 l of Tris-EDTA (TE) buffer. Following a five-minute centrifugation at 10,000 rpm, the waste product was removed, and the sample was two or three times cleansed with brand-new TE buffer. After removing the supernatant, 50 l of lysis buffer I had to be added, agitated, and left for five minutes. After aggressive vortexing, 50 µl of lysis buffer II was added. There was also the addition of 10 µl of proteinase K (10 mg/ml). It was then introduced to 60°C water in a water bath for two hours. After that, the enzymes were maintained in a water bath that was boiling for a period of 10 minutes. DNA-containing supernatant was transferred to a new tube and refrigerated at -20 degrees centigrade. The HPV 16, as well as 18 primers, were used in a standard PCR for the amplification.

Procedure for the polymerase chain reaction

For each sample, HPV 16 and HPV 18 were found using two different reactions. The following are the procedures to follow for preparing a PCR reaction mixture: PCR master mix is delicately agitated and centrifuged for a shorter duration after thawing. For each 50 µl PCR reaction, the subsequent ingredients are introduced to an ice-filled slim-walled PCR tube. Each of the tubes received an aliquot of a manufactured premix. The premix comprises the following ingredients with a total volume of 20 µl/aliquot. The specimens are softly spun down and agitated. After that, tubes are put in a standard heat cycler.

Here are the circumstances around the PCR: the first phase of denaturation took place for a duration of five minutes at a temperature of 95°C. The process of denaturation took place for a duration of one minute at a temperature of 95°C. The process of annealing took place for a duration of one minute at a temperature of 53°C and the process of extension was carried out for a duration of two minutes at a temperature of 72°C. The final extension took place for five minutes at 72°C. HPV 16 as well as HPV 18-specific patterns were found by placing the amplified products on 2% agarose gel. Different reactivity for HPV 16 and HPV 18 strains was done on different gels. Then, applying the gel documentation technology (Major Science, Taoyuan City, Taiwan), patterns were documented after a snapshot of the gel had been obtained underneath an ultraviolet light transilluminator. The HPV 16 amplicon is 120 base pairs in size. The remaining patterns were regarded as generic. A 100-base pair amplicon is equivalent to HPV 18. The remaining bands were regarded as generic.

Statistic evaluation

The Statistical Package for Social Sciences (SPSS) version 20.0 program (IBM Corp., Armonk, NY) was used to do statistical computation after the obtained data had been pasted into an Excel spreadsheet (Microsoft Corp., Redmond, WA). The chi-square test was used to compare the two subgroups HPV 16 and HPV 18, and HPV 16 positivity and HPV 18 positivity levels. Specifically, we used the chi-square test to compare the prevalence of HPV 16 and HPV 18 between the OSCC cases and the age-matched healthy controls. This involved cross-tabulating the presence or absence of each HPV variant within both groups and calculating the chi-square statistic. To determine statistical significance, a p-value threshold was chosen. In this study, the chosen threshold was p = 0.05. This means that if the p-value obtained from the chi-square test was less than 0.05, it would indicate a statistically significant difference in the prevalence of HPV variants between the two groups.

Ethical consideration

The ethical approval for the study was taken from the Srinivas Institute of Medical Sciences and Research Centre, Karnataka with institutional review board number (IRB) IEC/SIMS/2021/43.

## Results

It was observed that 22 cases out of 40 cases of OSCC were found positive for HPV-DNA. While 12 out of 40 age-matched healthy controls were found positive for HPV-DNA. On carrying out statistical analysis, the variation between the two categories was non-meaningful statistically (p = 0.367). However, the prevalence was greater in the case subgroup.

Out of 40 cases of OSCC, 12 cases were found positive for HPV 16. While six out of 40 age-matched healthy controls were found positive for HPV 16. On carrying out statistical analysis, the variation between the two categories was non-meaningful statistically (p = 0.114). However, the prevalence was greater in the case subgroup.

Out of 40 cases of OSCC, six cases were found positive for HPV 18. While two out of 40 age-matched healthy controls were found positive for HPV 18. On carrying out statistical analysis, the variation between the two categories was non-meaningful statistically (p = 0.236). However, the prevalence was greater in the case subgroup.

Four cases out of 40 cases of OSCC were found positive for HPV 16 and 18. While four out of 40 age-matched healthy controls were found positive for HPV 16 and HPV 18. On carrying out statistical analysis, the variation between the two categories was non-meaningful statistically (p = 0.662). However, the prevalence was greater in the case subgroup (Table 1).

**Table 1:** Prevalence of different strains of HPV in controls and cases HPV: human papillomavirus; DNA: deoxyribonucleic acid.

Variables	Cases (n = 40)	Control (n = 40)	P-value
HPV-DNA	22	12	0.367
HPV 16	12	6	0.114
HPV 18	6	2	0.236
Both HPV 16 and HPV 18	4	4	0.662

The gender distribution and age distribution has been documented in Table 2. It was observed that 10 out of 24 males in the case category were found to have positive HPV 16. Two males were found to be HPV 18 positive. It was found that both HPV 16 and HPV 18 were found in two males. Ten males were not found to have any strain of HPV 16 and HPV 18. It was observed that two out of 16 females were found to be HPV positive. Four females were found to be HPV 18 positive. Two females were found to be positive for both HPV 16 and HPV 18 strains. It was observed that eight females out of 16 females were not found to have any strain of HPV 16 or HPV 18.

**Table 2:** Evaluation of the prevalence of different strains of HPV among OSCC cases in relation to age and gender HPV: human papillomavirus; DNA: deoxyribonucleic acid; OSCC: oral squamous cell carcinoma; χ²: chi-squared; df: degrees of freedom.

Parameters	Gender		Age (years)			
	Male	Female	20-30	31-40	41-50	51-60
Total	24	16	6	4	10	20
HPV 16	10	2	2	2	2	6
HPV 18	2	4	2	0	0	4
Both	2	2	0	0	4	0
None	10	8	2	2	4	10
χ2	2.518		8.620			
df	4		10			
P	0.603		0.594			

Two participants in the case category in the age group of 20-30 years were found positive for HPV 16. Two participants were found positive for HPV 18. Two participants were not found positive for either HPV 16 or HPV 18. Two participants in the case category in the age group of 31-40 years were found positive for HPV 16. Two participants in the age group of 31-40 years were not found to be infected with either HPV 16 or HPV 18 variants.

Two participants in the case category in the age group of 41-50 years were found positive for HPV 16. Four participants were found to be infected with HPV 18 and HPV 16 variants. Four participants were not found infected with HPV 16 or HPV 18 variants.

Six participants in the case category in the age group of 51-60 years were found positive for HPV 16. Four participants in the case category were found positive for HPV 18. Zero participants in the case category were found infected with both HPV 18 and HPV 16 variants. Ten participants in the age group of 51-60 years were not found infected with HPV 16 or HPV 18 variants.

When there was an analysis of the correlation between the frequency of the HPV 16 variant and the HPV 18 variant between age and gender distribution, it was observed that the correlation was not meaningful statistically (p = 0.603 and p = 0.594; Table 2).

The prevalence of HPV different strains among participants of the case category in accordance with location and stage of differentiation has been shown in Table 3.

**Table 3:** Evaluation of frequency of different strains of HPV among OSCC patients in relation to location and stage of differentiation HPV: human papillomavirus; DNA: deoxyribonucleic acid; OSCC: oral squamous cell carcinoma; χ²: chi-squared; df: degrees of freedom.

Parameters	Location				Stage of differentiation		
	Posterior most area	Buccal mucosa	Tongue	Lower anterior area	Well-differentiated	Moderately	Basaloid
Total	18	10	10	2	34	4	2
HPV 16	2	4	2	2	12	0	0
HPV 18	2	2	2	0	4	0	2
Both	4	0	0	0	4	0	0
None	10	4	4	0	14	4	0
χ2	6.728				8.508		
df	10				7		
P	0.788				0.315		

It was observed that two out of 18 specimens from the posterior-most area were found to be infected with HPV 16. Two out of 18 specimens from the posterior-most area were found to be infected with HPV 18. Four out of 18 specimens from the posterior-most area were found to be infected with both HPV 18 and HPV 16 strains. Ten out of 18 specimens from the posterior-most area were not found to have any HPV strain.

It was observed that four out of 10 specimens from buccal mucosa were found to be infected with HPV 16. Two out of 10 specimens from buccal mucosa were found to be infected with HPV 18. Zero out of 10 specimens from buccal mucosa were found to be infected for both HPV 18 strain and HPV 16 strain. Four out of 10 specimens from buccal mucosa were not found to have any HPV strain.

It was observed that four out of 10 specimens from the tongue were found to be infected with HPV 16. Four out of 10 specimens from the tongue were found to be infected with HPV 18. Zero out of 10 specimens from the tongue were found to be infected with both HPV 18 and HPV 16 strains. Four out of 10 specimens from the tongue were not found to have any HPV strain. It was observed that two out of two specimens from the lower anterior region were found to be infected with HPV 16.

It was observed that 12 out of 34 specimens of the well-differentiated variant were found to be infected with HPV 16. Four were found to be infected with HPV 18. Four were found to be infected with HPV 18 and HPV 16. Fourteen were not found to be infected with HPV 18 or HPV 16.

It was observed that four out of four moderately differentiated variants were not found to be infected with HPV 18 or HPV 16. It was observed that two out of two basaloid differentiated variants were not found to be infected with HPV 18.

When there was statistical analysis, then it was found that the correlation between location and grade of differentiation of OSCC was not significantly associated with the prevalence of HPV (p = 0.788 and p = 0.315) (Table 3).

## Discussion

Compared to OPSCCs without HPV, which are typically associated with both alcohol consumption and cigarette addiction, OPSCCs with HPV represent a distinct clinical condition with a much better prognosis [19-21]. There is growing evidence that risky HPV types, primarily HPV 16/18, and OSCC are causally related.

The field of research into cancer in humans has expanded, thanks to the introduction of extremely accurate cellular biology techniques, particularly PCR, which enables virus identification immediately after invasion and often even before the onset of disease [22,23]. Utilizing PCR, the presentation of HPV variants like HPV 18 and HPV 16 in patients affected by OSCC was evaluated in this research.

It was observed in this research that 22 cases out of 40 cases of OSCC were found infected with HPV-DNA. While 12 out of 40 age-matched healthy controls were found infected with HPV-DNA. Out of 40 cases of OSCC, 12 cases were found infected with HPV 16. While six out of 40 age-matched healthy controls were found infected with HPV 16. Six cases out of 40 cases of OSCC were found infected with HPV 16. While two out of 40 age-matched healthy controls were found infected with HPV 18. Four cases out of 40 cases of OSCC were found infected with HPV 16. While four out of 40 age-matched healthy controls were found infected with HPV 16 and HPV 18. On carrying out statistical analysis, the variation between the two categories was non-meaningful statistically (p = 0.662). However, the prevalence was greater in the case (OSCC) subgroup.

In line with studies conducted by Gan et al. [20], who also noted that HPV proportion was greater among patients when compared to age controls, the results of the current investigation showed a higher proportion of HPV incidence among OSCC cases compared to controls. According to Zhu et al.'s [24] meta-analysis study examining the link between OSCC along HPV infection in the Chinese population, there are significant rates of HPV infection, especially HPV 16, which increases the risk of OSCC carcinogenesis. This is consistent with the results of our recent investigation, where a substantially greater prevalence of HPV 16 was noted. In a research investigation, D’Costa et al. [25] used PCR to find HPV 16 DNA and HPV 18 DNA in samples from people with cancer of the oral cavity, people with potentially cancerous lesions (PMLs), and those with HOM. Displaying that infections caused by HPV are crucial but may not be substantial for the development of cancers while suggesting that synergistic effects with other cancer-causing substances may be needed, they discovered HPV 16 infection in 15% of individuals with normal oral mucosa (NOM), 34% of PMLs, and 15% of OSCC.

In our study, 10 out of 24 males in the case category were found to have infected HPV 16. Two males were found to be HPV 18 infected. It was found that both HPV 16 and HPV 18 were found in two males. Ten males were not found to have any strain of HPV 16 and HPV 18. It was observed that two out of 16 females were found to be HPV infected. Four females were found to be HPV 18 infected. Two females were found to be infected with both HPV 16 and HPV 18. It was observed that eight females out of 16 females were not found to have any strain of HPV 16 or HPV 18.

This is consistent with studies done by Brandwein et al. [22] as well as Benson et al. [23] that found that men are more likely than women to have OSCC diagnosed.

It is believed that an important risk factor for the development of HPV-linked SCC of the head and neck is a person's lifetime total of sexual relationships. Sexual contact is how HPV, an infection, is spread [25-28]. According to case-control studies, those who admitted to having between one and five oral sex relationships in their lifetimes were twice as likely to develop a malignancy with HPV positivity, and those who admitted to having six or more partners were five times more likely [18]. There is a wide range of variation in the rates of HPV positivity in malignancies at various sites in the HNR [23]. The most common cancers are those of the tonsils and buccal mucosa, tongue, and floor of the mouth.

It was observed that two out of 18 specimens from the posterior-most area were found to be infected with HPV 16. Two out of 18 specimens from the posterior-most area were found to be infected with HPV 18. Four out of 18 specimens from the posterior-most area were found to be infected with both HPV 18 strain and HPV 16. It was observed that four out of 10 specimens from buccal mucosa were found to be infected with HPV 16. Two out of 10 specimens from buccal mucosa were found to be infected with HPV 18. It was observed that four out of 10 specimens from the tongue were found to be infected with HPV 16. Four out of 10 specimens from the tongue were found to be infected with HPV 18. It was observed that two out of two specimens from the lower anterior region were found to be infected with HPV 16.

In a study by Paz et al., to determine the relationship between HPV 16 and SCC, HPV sequences were discovered in 15% of tumors, while Waldeyer's tonsillar ring tumors had the highest HPV detection rate [27].

The Epstein-Barr virus, the HPV, and the HSV-1 are additional viruses that may aid in the development of OSCC [16]. Humans' various epithelial tissues are invaded by smaller DNA viruses known as HPV.

It was observed that 12 out of 34 specimens of the well-differentiated variant were found to be infected with HPV 16. Four were found to be infected with HPV 18. Four were found to be infected with HPV 18 and HPV 16. Fourteen were not found to be infected with HPV 18 or HPV 16. It was observed that four out of four moderately differentiated variants were not found to be infected with HPV 18 or HPV 16. It was observed that two out of two basaloid differentiated variants were not found to be infected with HPV 18.

According to histology, HPV head and neck squamous cell carcinomas (HNSCCs) are non-keratinizing with basaloid characteristics, according to Benson et al.'s 2014 report [23]. They were once assumed to be weakly differentiated, but after more examination, it was shown that they share morphology with the tonsillar crypts, which are where they are thought to originate; therefore, it is more accurate to describe them as well-differentiated [23].

The clinical implications of this observation are noteworthy. Well-differentiated OSCC variants with HPV involvement may represent a distinct subgroup within oral cancer patients. Understanding the histological and molecular characteristics of these tumors is essential for tailoring treatment strategies. Although HPV-positive HNSCCs, including OSCC, have been associated with a better prognosis compared to their HPV-negative counterparts, the specific impact of histological differentiation on treatment response and patient outcomes warrants further investigation. It is conceivable that well-differentiated OSCC cases with HPV infection may respond differently to treatment modalities such as surgery, radiation therapy, or chemotherapy. Therefore, clinicians should consider histological differentiation and HPV status as potential prognostic indicators when developing individualized treatment plans. Additionally, ongoing research into the molecular pathways and biological behavior of well-differentiated HPV-related OSCC can provide insights into the mechanisms underlying their unique clinical characteristics.

The study had some limitations. The sample size in the study was relatively small, consisting of 40 cases of OSCC and 40 age-matched healthy controls. A larger and more diverse sample size might have provided more statistically robust results, allowing for greater generalizability of the findings to the broader population. Additionally, the study's cross-sectional design captured data at a single point in time. Longitudinal studies that track individuals over an extended period could offer deeper insights into the relationship between HPV infection and the development of OSCC. Such studies could help establish causal links and assess the progression of HPV-related carcinogenesis. Selection bias may have been introduced as cases and controls were selected based on specific criteria. Employing a more random or representative sampling method could enhance the study's external validity and reduce potential bias. Addressing these limitations and conducting further research with a larger, more diverse sample and comprehensive clinical data can provide a deeper and more accurate understanding of the role of HPV in the development of OSCC.

## Conclusions

When evaluated against controls in the current investigation, OSCC cases had a greater level of HPV expression and a greater proportion of HPV 16 positives. However, there was no statistically noteworthy change. The majority of cases showing HPV positive corresponded to a well-differentiated OSCC subtype. Despite the fact that patients had more HPV than controls did, neither of those variations was statistically noteworthy. Because HPV 16 and HPV 18 variants are frequently discovered in NOM, it is essential to distinguish between clinical HPV infections, subclinical infections, and latent infections. As a result, conclusive findings require additional research on bigger samples utilizing sensitive detection methods like real-time PCR.
